# The Roles of CD147 and/or Cyclophilin A in Kidney Diseases

**DOI:** 10.1155/2014/728673

**Published:** 2014-12-17

**Authors:** Xin Qu, Chunting Wang, Jicheng Zhang, Guoqiang Qie, Jianxin Zhou

**Affiliations:** ^1^Department of Critical Care Medicine, Shandong Provincial Hospital, Shandong University, 324 Jingwu Road, Jinan 250021, China; ^2^Department of Critical Care Medicine, Beijing Tiantan Hospital, Capital Medical University, 6 Tiantan Xili, Beijing 100050, China

## Abstract

CD147 is a widely expressed integral plasma membrane glycoprotein and has been involved in a variety of physiological and pathological activities in combination with different partners, including cyclophilins, caveolin-1, monocarboxylate transporters, and integrins. Recent data demonstrate that both CyPA and CD147 significantly contribute to renal inflammation, acute kidney injury, renal fibrosis, and renal cell carcinoma. Here we review the current understanding of cyclophilin A and CD147 expression and functions in kidney diseases and potential implications for treatment of kidney diseases.

## 1. Introduction

CD147 is a ubiquitously distributed integral transmembrane glycoprotein belonging to the immunoglobulin (Ig) superfamily [1]. And it has been implicated in a number of physiological and pathological effects through interacting with different binding partners such as cyclophilins (CyPs), caveolin-1, monocarboxylate transporters, integrins, and E-selectin [2]. To date, lots of studies have demonstrated that CD147 has taken part in the regulation of lymphocyte responsiveness, carcinoma metastasis, monocarboxylate transporter (MCT) induction, inflammatory responsive, and spermatogenesis [3]. Among these partners, cyclophilins, especially cyclophilin A (CyPA), might be investigated most frequently in the recent years. CyPA is a ubiquitously distributed protein that belongs to the immunophilin family which share peptidyl-prolyl* cis-trans* isomerase activity [4, 5]. Current research has provided compelling evidences to identify the key function of CyPA in several human diseases such as viral infections, cardiovascular diseases, cancer, rheumatoid arthritis, sepsis, and asthma [4]. Expression of CD147 on the renal tubular cells was reported in chickens [6] and rabbits [7] for the first time. In 2009, Shimada et al. initially observed that CD147 was diffusely expressed in the proximal and distal tubular epithelial cells of most patients and healthy adults but was not detected in glomeruli [8]. Nowadays, a growing body of research suggested CyPA and CD147 involvement in key processes of kidney disease pathologies. The objective of this paper is to review the current knowledge of CyPA and CD147 regarding potential roles in kidney diseases to offer novel therapeutic strategies.

## 2. Expression and Function of CyPA

CyPs are a family of ubiquitously distributed proteins that are evolutionarily well conserved and exist in all cells of organisms in both prokaryotes and eukaryotes [4]. Human CyPs contain 16 family members which are structurally different and located intracellularly as well as extracellularly [4]. Among these family members, CyPA which is a primarily intracellular protein and the founding number of CyPs is expressed abundantly in all mammalian cell types. CyPA was first purified from bovine thymocytes in 1984 and confirmed as the primary intracellular receptor of the immunosuppressive drug cyclosporin A (CsA) [9, 10]. Among these known human CyPs, CyPA as a housekeeping protein is the most abundant cytosolic member, which accounts for ~0.1–0.6% of the complete intracellular proteome [9–11]. CyPA gene is localized to the region 7p11.2-p13 [10, 12]. The structure of human CyPA contains eight strands of antiparallel *β*-sheets in a flattened *β*-barrel with two helices capping the top and bottom [13]. Although CyPA is primarily located intracellularly, it can be secreted into the extracellular environment in various cell types due to inflammatory stimuli such as infection, hypoxia, and oxidative stress [11, 14–16]. The concrete mechanism of the CyPA-release in these cells presumably might be associated with the acetylation of CyPA [17]. Furthermore, acetylated CyPA seems to play a more significant inflammatory role than unmodified CyPA in vascular smooth muscle cells [17]. The secreted form of CyPA known as an autocrine/paracrine factor may mediate intercellular signal communication and is identified to be a potent chemoattractant for monocytes [18], neutrophils [18, 19], eosinophils [19], and T cells [20]* in vitro*. At present, some research confirmed CD147 as a surface receptor for extracellular CyPA [21]. The chemotactic activity of CyPA is mediated, in part, through binding with CD147 receptor [21]. In addition, similar to other cyclophilins, CyPA possesses an activity of peptidyl-prolyl* cis-trans* isomerase which catalyzes the isomerization of peptide bonds from* trans* form to* cis* one at proline residues to prompt protein folding [4, 22] and may play crucial roles in many biological conditions including protein folding, trafficking, assembly, T cell activation, and cell signaling [4, 23]. CyPA pertains to a diverse set of proteins known as molecular chaperones due to its cellular localization, enzymatic properties, and role in protein folding [24]. The increased levels of soluble extracellular CyPA can be detected in patients with inflammatory responses such as in serum of patients with sepsis [25], in nasal fluids of patients with asthma [26], and in plasma of patients with coronary artery disease [27]. Some studies with mutant CyPA proteins demonstrate that CyPA can induce chemotaxis of leukocyte and signalling via two distinct pathways: PPIase activity [21] and extracellular binding to CD147 [13]. Some research with NMR has demonstrated that CyPA efficiently catalyzes prolyl* cis-trans* isomerization of cell signaling adaptor protein Crk, HIV-1 capsid protein, and interleukin-2 tyrosine kinase and thus modulates their functions [13]. The detailed functions of CyPA in various types of cells are needed to be further studied. Furthermore, CyPA was reported to be implicated in kidney epithelial differentiation via the hensin polymerization pathway. Hensin, which is a multidomain, multifunctional 230-kDa extracellular matrix protein, is a rabbit ortholog of the human DMBT1 gene and is involved in the modulation of epithelial differentiation, innate immunity defense, and tumorigenesis [28]. Hensin expression in most epithelia is detected in various alternately spliced forms. Peng et al. observed that cyclosporin A, the inhibitor of CyPA, modulates the extracellular matrix assembly of hensin and the differentiation of kidney epithelial cells by suppressing PPIase activity of CyPA [28] and thus demonstrated a direct impact of CyPA-mediated PPIase activity on kidney epithelial differentiation for the first time. The results suggest that PPIase activity of CyPA could regulate kidney epithelial differentiation by the hensin polymerization.

CyPA was reported to be a crucial proinflammatory signaling pathway in monocytes [29, 30]. Wei et al. illustrated that CyPA stimulation activated Akt and NF-*κ*B signaling pathways and therefore elevated antiapoptotic protein Bcl-2 expression in endothelial cells [30]. They also observed that CyPA treatment activated NF-*κ*B by ERK1/2 pathway in human monocytic cell line THP-1 [29]. Several studies demonstrated that secreted CyPA could bind to and activate the cell surface receptor CD147 and then result in increased ERK and Akt signaling [10, 21, 31]. Therefore, to date, the main signaling pathways associated with CyPA/CD147 include Akt, ERK1/2, MAPK, and NF-*κ*B.

## 3. Expression and Function of CD147

CD147 is a highly glycosylated transmembrane protein belonging to the immunoglobulin superfamily and is encoded in human by a gene localized to 19p13.3 [32]. And the human CD147 gene locus has 10 exons [33]. Four splice variants, named CD147-1, CD147-2, CD147-3, and CD147-4, are transcribed from the human CD147 gene on the basis of data in the Entrez Gene Database. Among these isoforms, CD147-2 is the most abundantly expressed and widely distributed variant of CD147, and, therefore, this form is designated as CD147. CD147 is extensively distributed at varying levels on the surface of various types of cells, including haematopoietic cells, epithelial cells, endothelial cells, immune cells, smooth muscle cells, and tumor cells [34–37]. It has different names in different species such as rats and chickens (HT7 [38] neurothelin and 5A11 antigen [39]) (OX-47 antigen [34] and CE9 [40]), human and mice (gp42 [41] and basigin [42]). CD147 is most recently described to induce the production of several matrix metalloproteinases (MMPs), leading to its renaming to EMMPRIN for “extracellular matrix metalloproteinase inducer” [37]. CD147 was demonstrated to have the structure Gal*β*1 → 4 (Fuc*α*1 → 3) GlcNAc, which is called the Lewis X structure [43]. In humans, this protein was first described by Biswas and colleagues as a tumor cell-derived collagenase-stimulatory factor termed TCSF made by tumour cells that stimulates production of a collagenase (matrix metalloproteinase type 1, MMP-1) by fibroblasts [44]. CD147 protein with different origins from human cells and tissues has been identified by various different laboratories [45] and has been named as an extracellular matrix metalloproteinase inducer (EMMPRIN) [35], HAb18G [46], or M6 antigen [47]. Human CD147 protein is composed of 269 amino acids, which constitute an extracellular domain containing 206 aa, a transmembrane domain containing 24 aa, and a cytoplasmic domain containing 39 aa [3, 48]. The transmembrane domain contains a leucine zipper and a charged residue (glutamic acid). The extracellular domain has three N-linked glycosylation sites, which proffer attachment sites to highly branched sugars, and glycosylation of these sites varies in the different organ. The differences of this glycosylation may result in a variety of physiological roles of CD147.

**Figure 1:**
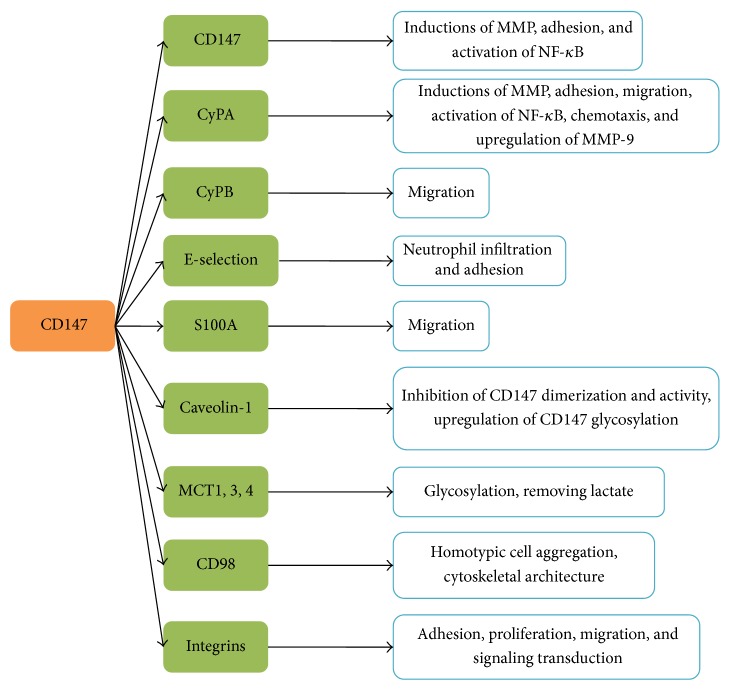
Illustration of CD147-interacting proteins and functions. MCT: monocarboxylate transporter; MMP: matrix metalloproteinase.

To our knowledge, CD147 exerts various roles through interacting with different ligands. Yurchenko et al. were the first to confirm CD147 as the principal signalling receptor for extracellular cyclophilins [21]. A large number of evidences have also identified CD147 as the principal cell surface receptor for introduction of Cyps signals into target cells [49]. CD147 binding partners are not fully known yet and the number is likely to grow in the future. Currently, besides the above mentioned, CD147 binding partners include cyclophilins, caveolin-1, monocarboxylate transporters (MCT-1,3,4) [50], integrins [51], E-selectin [2], S100A9, CD98, CD44, and CD147 itself [52] (Figure 1). The extracellular domain of CD147 was demonstrated to interact with caveolin-1, cyclophilins, *β*1 integrin, and CD147 itself, and the transmembrane domain is associated with CD43, MCT, and syndecan [43, 53]. The ligands including caveolin-1 and E-selectin are simply introduced as follows.

### 3.1. E-Selectin

E-selectin (endothelial selectin), an adhesion molecule, is one of the selectin family with well-characterized roles in leukocyte homing [54]. It is typically expressed by the endothelium at sites of injury or inflammatory stimulation. Kato et al. observed in mice with renal ischemia/reperfusion injury that CD147^−/−^ neutrophils showed less binding to E-selectin. And they also found by injection of labeled neutrophils into mice that CD147^−/−^ neutrophils were less readily recruited to the kidney than Bsg^+/+^ ones. These results suggest that CD147 is also a physiologic ligand for E-selectin and plays an indispensable effect on adhesion to vascular endothelial cells in the renal damage caused by ischemia/reperfusion. The effect might be associated with sialyl Lewis X in the structure of CD147 as a minimal recognition motif for E-selectin. Besides CD147, three representative E-selectin ligands on neutrophils including P-selectin glycoprotein ligand-1 (PSGL-1), E-selectin ligand-1, and CD44 have been identified [2].

### 3.2. Caveolin-1

Caveolin-1 is a pivotal modulator of caveolae-dependent signaling and a principal component of plasma membrane caveolae [55]. Caveolin-1 has been demonstrated to directly interact with CD147 [56]. Tang and Hemler observed that caveolin-1 could prevent CD147 formation to reduce MMP production [57] and presumed that CD147 might be negatively regulated by caveolin-1. Conversely, there is evidence that overexpression of caveolin-1 could result in an increase of highly glycosylated form of CD147 and facilitate cell invasion by inducing MMP production in murine hepatocarcinoma cell lines [58].

Upregulation of CD147 has been involved in the pathogenesis of a number of diseases, such as asthma [26, 59], lung inflammation [60], rheumatoid arthritis [52, 61], coronary artery disease [62, 63], and tumors [63, 64]. Elevated CD147 levels were also observed in a great number of malignant tumours and have been found to be associated with tumour development and progression in experimental and clinical conditions. CD147 also prompts viral infection and the invasion of some microorganisms into host cells [1, 65]. Additionally, a growing body of evidence suggests that CD147 performs various functions in both membrane-bound and soluble forms [66]. Signalling pathways via CD147 comprise activation of PI3-kinase, ERK1/2, MAPK, and nuclear factor kappa B in a cell-dependent manner [67, 68].

At present, the precise molecular mechanisms of CyPA/CD147 interaction have still not been illuminated in detail. Yurchenko et al. presumed that the enzymatic activity of CyPA is required for CD147-mediated signalling on the basis of the experiments with mutants of CyPA without PPIase activity [21]. Furthermore, Seizer et al. demonstrated that the CD147-binding site of CyPA overlaps with the PPIase active site [11]. Importantly, they also found that mutants of CyPA, with a conserved CD147-binding site and without enzymatic activity, still produced a strong chemotactic effect, suggesting that the chemotactic activity of CyPA could be directly mediated via binding to CD147 [11, 13].

In addition, CD147 deficiency, CyPA deficiency, or anti-CD147 monoclonal antibody was found to substantially reduce the infarct size at 24 hours and 7 days in acute myocardial infarction after ischemia/reperfusion [69]. The results might be associated with reduced monocyte and neutrophil recruitment. Furthermore, consistent with the previous study [60], treating with a combination of anti-CyPA and anti-CD147 did not create a better protective effect as compared to the individual treatments [69], suggesting that anti-CD147 and anti-CyPA might act on the same cyclophilin-CD147 interaction.

## 4. CD147 and CyPA in Renal Cell Carcinoma

Several studies of CD147 in tumor tissues demonstrated that upregulated expression is associated with clinically aggressive behavior and poor prognosis in a variety of cancer types [70, 71]. Renal cell carcinoma (RCC) which represents the most common malignancy of kidneys found in adults makes up 2-3% of all malignant tumors in adults [72]. In several years, the functions of CD147 in RCC have been evaluated using many experimental methods. In 2006, Tsai et al. initially demonstrated that CD147 and MMP-9 were overexpressed in renal cell carcinomas by immunohistochemistry and upregulation of CD147 in tumor cells was associated with poor prognosis of patients with clear cell renal cell carcinoma (CRCC) [73]. Besides, they also found that higher expression levels of CD147 were correlated with higher T staging and nuclear grading in CRCC. Tsai et al. demonstrated that overexpression of CD147 and fascin in RCC correlated positively with advanced clinical stages and survival time and higher CD147 immunoscores also correlated positively with fascin in RCC [74]. So they speculated that anti-CD147 antibody might be effective in inhibiting tumor growth and development of multidrug-resistant RCC. But they did not test the hypothesis. Later, Liang et al. reported that CD147 and VEGF were overexpressed in most of the patients with advanced RCC, and both were significantly correlated with TNM stage and prognosis of advanced RCC [75]. Additionally, they confirmed that conjoined expressions of CD147^−^/VEGF^−^ and CD147^+^/VEGF^+^ were independent prognostic indicators of advanced RCC (both *P* < 0.01). In 2013, Sato et al. observed* in vitro* and* in vivo* that downregulation of CD147 by siRNA resulted in decreased VEGF and bFGF expression, cell proliferation, and invasive potential, and overexpression of CD147 was observed in patients who received sunitinib therapy as well as in sunitinib-resistant 786-O cells [64]. Based on the above studies, we might draw conclusions that CD147 could play an important role in the progression of advanced RCC and CD147 could be a novel target for the treatment of RCC.

Among the members of the CyPs family, CyPA was first demonstrated to be overexpressed in tumours, such as pancreatic cancer and breast cancer [76]. CyPA in cancer biology interacts with CD147, which was first confirmed in human pancreatic cancer in 2006 [77]. Yurchenko et al. demonstrated that CyPA regulated the cell surface expression of CD147 via the transmembrane domain of CD147 [78], thereby facilitating the pancreatic cancer cell proliferation. The overexpression of CyPA in many kinds of cancers correlates closely with tumour development, malignant transformation, proliferation, apoptosis, and metastasis [76]. It is speculated that CyPA might exert pivotal roles in the development and prognosis of RCC and might be a novel therapeutic target for RCC. However, the above research on CyPA and the molecular mechanism of CD147 is given less concern. Therefore, more research is needed to identify the function of CypA and the molecular mechanism of CD147 and/or CypA in renal cell carcinoma.

## 5. CD147 and CyPA in Acute Kidney Injury

Acute kidney injury (AKI) has been estimated to account for 1~2% of hospital inpatients and the prevalence is more than 40% at admission to the intensive-care unit [79, 80]. And mortality in ICU patients with AKI and multiorgan failure has been reported to be greater than 50% [81]. The mechanism of AKI remains unclear. In 2009, Dear and his colleagues [82] demonstrated in cecal ligation and puncture (CLP) induced organ dysfunction by difference in-gel electrophoresis (DIGE) that cyclophilins including CyPA increased in abundance after CLP and sepsis-induced renal dysfunction was significantly attenuated after CD147, the receptor for CyPA, was inhibited by anti-CD147 antibody intraperitoneally. Furthermore, serum TNF-*α*, IL-6, and IL-10 concentration 24 hrs after CLP significantly decreased, which suggested that injection of anti-CD147 antibodies significantly reduced pro- and anti-inflammatory cytokine production. Therefore, the authors concluded that anti-CD147 could prevent AKI and CD147 would seem to be a novel target for sepsis-induced AKI, which is consistent with the notion of Kato et al. [2]. CD147 gene-deficient mice also showed less tubular injury by preventing neutrophil migration after renal ischemia/reperfusion which is one of the principal mechanisms of AKI [2].

To our knowledge, energy consumption might be associated with AKI [83]. Recently, CD147 deficiency is found to induce ATP depletion in AKI caused by ischemia and the primary cultured Bsg^−/−^ TECs result in ATP depletion by hypoxia [43]. These results imply that CD147 might participate in the lactate metabolism cycle through interaction with MCT, which is one of the functions of CD147 in AKI.

**Figure 2:**
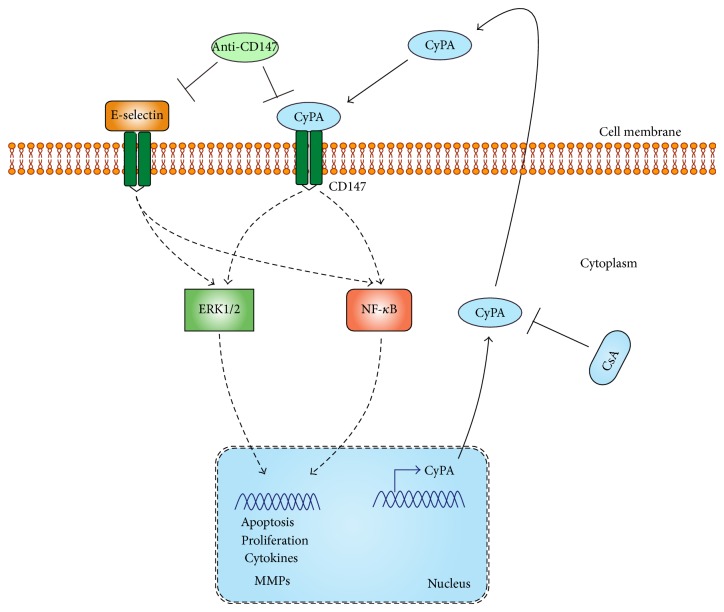
A schematic representation of a proposed mechanism implicated in CyPA/CD147-mediated cell response in AKI. CyPA is overexpressed after CLP and renal dysfunction is significantly attenuated by anti-CD147 antibody intraperitoneally. Specific inhibitor against CyPA might also improve the renal function. CD147 may result in activation of ERK1/2 and NF-kB, involved in cell apoptosis, proliferation, and the product of cytokines and MMPs.

Several studies have demonstrated that extracellular cyclophilin exerts proinflammatory effect via CD147 and anti-CD147 antibodies are anti-inflammatory (Figure 2) [59, 84]. The pathophysiological relevance of CyPA-CD147 interaction for inflammatory processes has been investigated in a number of animal models. The research on synovial macrophages of rheumatoid arthritis patients observed that CyPA and CD147 expression were detected and stimulation of CD147 induced the production of MMP-9 and proinflammatory cytokines and promoted cell migration in macrophages. Consequently, blocking the interaction between CD147 and CyPA by antibodies in a collagen-induced arthritis model led to a significant reduction in arthritic symptoms [85]. However, as far as ischemic renal injury is concerned, CD147 might be a double-edge sword in the process of diseases, because upregulated CD147 might result in tissue destruction through elevating MMP production and inducing leukocyte infiltration in ischemic tissue [55]. Recently, CD147 was found to interact with E-selectin to promote renal inflammation in renal ischemia/reperfusion injury by increasing neutrophil infiltration into the tubulointerstitium [2], which might play a crucial role in the pathogenesis of postischemic renal failure through releasing cytotoxic proteases and oxygen-derived radicals. Nevertheless, there were no differences in CyPA expression between wild-type and Bsg^−/−^ mice with ischemic AKI, suggesting that the interaction between CD147 and CyPA might not be involved in the development of ischemic AKI [43]. Further studies are required to fully understand this interaction.

More recently, Zhu et al. suppose through investigating CD147 in hepatocellular carcinoma that more CD147 expression might arouse the response of inflammatory cytokines and T cells to improve the immune environment to delay the progression of hepatocellular carcinoma [86], similar to the possible roles of CD147 in sepsis. At present, our team is investigating the potential roles and mechanisms of CD147/CyPA in sepsis and sepsis-induced AKI. The studies in the future will hopefully integrate these findings into diagnosis and/or treatment of sepsis and sepsis-induced AKI.

## 6. CD147 and CyPA in Nephritis

Lupus nephritis (LN) usually results in higher mortality and poor quality of life. The diagnostic accuracy for many biomarkers, such as neutrophil gelatinase-associated lipocalin (NGAL) and monocyte chemoattractant protein-1 (MCP-1), remains unsatisfactory [87]. CD147 was supposed to be complicated in the pathogenesis of LN through the polarization of T lymphocytes. More recently, a study was performed to observe that CD147 was strikingly expressed in injured glomeruli and infiltrating inflammatory cells without injured tubules, and both plasma and urinary CD147 levels in patients with LN were double those of pathological control ones and healthy controls [87]. It had also been reported that plasma CD147 levels correlated strongly with urinary CD147 levels and serum Cr values, whereas urinary levels of CD147 were markedly elevated than plasma ones. Plasma CD147 appeared to be significantly overexpressed in LN with severe inflammation and positively correlated significantly with renal disease activity, suggesting that plasma CD147 levels could reflect the disease activity of LN. In addition, the combination of plasma CD147 and component C3 obtained a reliable AUC level (AUC, 0.92) for estimating pathological LN activity and producing accurate diagnosis for guiding ideal LN therapy. As for CyPA, it participates in inflammatory response, and, thus, it might be associated with LN.

## 7. CD147 and CyPA in Renal Fibrosis

Renal fibrosis is the important determinant and prognostic predictor of chronic progressive kidney failure, which accounts for approximately 8–10% of individuals in the developed countries [88–90]. The precise cellular mechanisms promoting tubulointerstitial fibrosis after acute kidney injury remain poorly eliminated [91]. Currently, it is considered that excessive accumulation of extracellular matrix (ECM) results in state of fibrosis [92]. ECM synthesis and degradation are complicatedly modulated by TGF-*β*, MMPs, and others.

A large body of research has shown that macrophage infiltrating into injured interstitium could increase the product of cytokines related to fibroblast proliferation and activation. As previously mentioned, CD147 is expressed on macrophages and acts as a ligand of E-selectin. Mice with a triple knockout of E-, P-, and L-selectin indicate a significant reduction in macrophage recruitment after unilateral ureteral obstruction surgery [93]. The data propose that CD147 expression on macrophages might exert a crucial role in macrophage infiltration into the interstitium through binding to selectins. In addition, an experimental study observed on tubular epithelial cells (TECs) that CD147 on fibroblasts is involved in the induction of hyaluronan, which is associated with fibroblast differentiation in response to TGF-*β* [43].

It is well known that CD147 induces the production of MMPs and CD147 expression in fibroblasts is increased by TGF-*β*1 to induce fibroblast-to-myofibroblast differentiation and to elevate cell contractility in tissue remodeling [94]. Therefore, it was proposed that CD147 could exert a pivotal role in the renal fibrosis. A study with mouse liver fibrosis model reported that the expression level of CD147 increased with the development of fibrogenesis and decreased during the liver fibrosis spontaneous recovery [94]. And the researchers found that CD147 antibody would exert inhibition effects on hepatic stellate cells activation and suppress the liver fibrosis [4]. A recent study revealed that Bsg^−/−^ mice in the interstitium using a unilateral ureteral obstruction model had much less fibrosis than Bsg^+/+^ ones at 14 days after operation [95], and MMP-2 and MMP-9 activities were suppressed in Bsg^−/−^ mice. Furthermore, TGF-*β* response was lower in primary Bsg^−/−^ tubular epithelial cells. The above-described results demonstrated that CD147 promotes the formation of renal fibrosis and suggested that CD147 would have multiple effects on promoting renal fibrosis such as the hyaluronan production, the regulation of MMPs, and macrophage infiltration and might be a novel candidate target gene for the prevention of organ fibrosis and therapeutics targeting. In the future, blockade of CD147 by siRNA or anti-CD147 antibody could be performed* in vivo and in vitro* to further verify the effects of CD147 on renal fibrosis.

CyPA and CD147 were found to be upregulated in patients with inflammatory cardiomyopathy. CyPA^−/−^ mice with coxsackievirus B3-induced myocarditis demonstrated a significantly reduced T cell and macrophage recruitment at 8 days compared with wild-type mice [96]. Treatment with NIM811, a CyPA inhibitor, led to a more significant reduction in myocardial fibrosis on day 12. The above study illustrates that CyPA might be involved in the formation of myocardial fibrosis. In a rat model with carbon tetrachloride-induced liver fibrosis, NIM811 was confirmed to decrease the expression of tissue inhibitor of metalloproteinase-1 and TGF-*β* [97]. Therefore, it is reasonable to assume that CyPA also participated in the formation of renal fibrosis.

## 8. Conclusion and Future Directions

Based on the above various studies reviewed, it is clear in kidney diseases that both CD147 and CyPA have multifunctional properties, both independently and as an interacting complex. Increasing evidence demonstrates a crucial role of CD147 in kidney diseases including renal carcinoma and AKI probably through regulating different cell signal pathways. Therefore, targeting CyPA and CD147 appears to represent a promising strategy for treating kidney diseases. siRNA specific targeting CD147 is a useful tool to silence CD147 gene and inhibit its activity [55]. However, the method had not been employed in the above studies. To illuminate particular functions of CD147/CyPA in kidney disease, more research is needed to perform to better understand the mechanisms underlying CD147/CyPA under different biological and pathophysiological conditions. Finally, it will be exciting to see if these findings could be exploited therapeutically and lead to the development of new drugs that prevent or possibly reverse established kidney diseases.
